# Wound contraction and macro-deformation during negative pressure therapy of sternotomy wounds

**DOI:** 10.1186/1749-8090-5-75

**Published:** 2010-09-30

**Authors:** Christian Torbrand, Martin Ugander, Henrik Engblom, Håkan Arheden, Richard Ingemansson, Malin Malmsjö

**Affiliations:** 1 Department of Ophthalmology, Lund University and Skåne University Hospital, Lund, Sweden; 2 Department of Clinical Physiology, Lund University and Skåne University Hospital, Lund, Sweden; 3 Department of Cardiothoracic Surgery, Lund University and Skåne University Hospital, Lund, Sweden

## Abstract

**Background:**

Negative pressure wound therapy (NPWT) is believed to initiate granulation tissue formation via macro-deformation of the wound edge. However, only few studies have been performed to evaluate this hypothesis. The present study was performed to investigate the effects of NPWT on wound contraction and wound edge tissue deformation.

**Methods:**

Six pigs underwent median sternotomy followed by magnetic resonance imaging in the transverse plane through the thorax and sternotomy wound during NPWT at 0, -75, -125 and -175 mmHg. The lateral width of the wound and anterior-posterior thickness of the wound edge was measured in the images.

**Results:**

The sternotomy wound decreased in size following NPWT. The lateral width of the wound, at the level of the sternum bone, decreased from 39 ± 7 mm to 30 ± 6 mm at -125 mmHg (p = 0.0027). The greatest decrease in wound width occurred when switching from 0 to -75 mmHg. The level of negative pressure did not affect wound contraction (sternum bone: 32 ± 6 mm at -75 mmHg and 29 ± 6 mm at -175 mmHg, p = 0.0897). The decrease in lateral wound width during NPWT was greater in subcutaneous tissue (14 ± 2 mm) than in sternum bone (9 ± 2 mm), resulting in a ratio of 1.7 ± 0.3 (p = 0.0423), suggesting macro-deformation of the tissue. The anterior-posterior thicknesses of the soft tissue, at 0.5 and 2.5 cm laterally from the wound edge, were not affected by negative pressure.

**Conclusions:**

NPWT contracts the wound and causes macro-deformation of the wound edge tissue. This shearing force in the tissue and at the wound-foam interface may be one of the mechanisms by which negative pressure delivery promotes granulation tissue formation and wound healing.

## Introduction

Cardiac surgery is complicated by post-sternotomy mediastinitis in 1% to 5% of all procedures [1] and is a life-threatening complication [2]. The reported early mortality in post-sternotomy mediastinitis following coronary artery bypass graft surgery is between 8% and 25% [3,4]. Conventional treatment of post-sternotomy mediastinitis includes surgical debridement, drainage, irrigation, and reconstruction using pectoral muscle flap or omentum transposition. In 1999, Obdeijn and colleagues described a new method of treatment for post-sternotomy mediastinitis using a vacuum-assisted closure technique [5], which is based on the principle of applying subatmospheric pressure by controlled suction through a porous dressing. The technique, also known as negative pressure wound therapy (NPWT), has resulted in reduced mortality in post-sternotomy mediastinitis [6].

Scientific evidence regarding the mechanisms by which NPWT promotes wound healing has started to emerge. NPWT results in the drainage of excessive fluid and debris, removal of wound edema, reduction in bacterial counts and stimulation of wound edge microvascular blood flow [7-10]. However, it is now believed that one of the major driving forces that generate granulation tissue formation is the macro-deformation of the wound edge tissue that results from the suction force created by the negative pressure. To our knowledge, there is only sparse scientific evidence for this instantaneous mechanical effect by NPWT [11].

The present study was performed to in detail investigate the effects of NPWT on wound contraction and wound edge tissue deformation. Magnetic resonance imaging (MRI) of the thorax was performed in a porcine sternotomy wound model. The lateral width of the wound and anterior-posterior thickness of the wound edge was measured in the images taken before and after initiation of NPWT at -75, -125 and -175 mmHg.

## Materials and methods

### Animals

An uninfected porcine sternotomy wound model was used in the present study. Six domestic landrace pigs of both genders, with a mean body weight of 50 kg, were fasted overnight with free access to water. The study was approved by the Ethics Committee for Animal Research, Lund University, Sweden. The investigation complied with the "Guide for the Care and Use of Laboratory Animals" as recommended by the U.S. National Institutes of Health and published by the National Academies Press (1996).

### Anesthesia

Anesthesia was induced with ketamine hydrochloride (Ketaminol Vet™ 100 mg/ml, Farmaceutici Gellini S.p.A, Aprilia, Italy), 15 mg/kg intramuscularly, and xylazine (Rompun Vet™ 20 mg/mL, Bayer AG, Leverkusen, Germany), 2 mg/kg intramuscularly. The pigs were intubated and mechanical ventilation was established with a Siemens-Elema 900B ventilator in the volume-controlled mode. Anesthesia was maintained by continuous intravenous infusion of propofol (Diprivan™, Astra Zeneca, Sweden), 0.1-0.2 mg/kg/min, in combination with fentanyl (Leptanal™, Lilly, France), 0.05 μg/kg/min, and atracurium besylate (Tracrium™, Glaxo, Täby, Sweden), 0.2-0.5 mg/kg/hour.

### Surgical procedure

After a midline sternotomy, the pericardium was opened and a polyurethane foam dressing was placed between the sternal edges. Two non-collapsible drainage tubes were inserted into the foam. The open wound was then sealed with a transparent adhesive drape. The drainage tubes were connected to a purpose-built vacuum source (VAC^® ^pump unit, KCI, Copenhagen, Denmark), which was set to deliver a continuous negative pressure of -75, -125 or -175 mmHg.

### Experimental procedure

MRI was first performed at baseline (0 mmHg). A negative pressure was then applied and MRI was performed when the target pressure had been reached. This procedure was repeated for each negative pressure (-75, -125, and -175 mmHg). In order to eliminate time effects, the sequence of application of the three different negative pressures was varied between the animals using a 3 by 3 Latin square design.

### Magnetic resonance imaging

MRI was conducted using a 1.5T system (Intera CV, Philips Medical Systems, Best, the Netherlands) with a five-element cardiac coil and the pig in the supine position. The images were acquired during ventilator-controlled end expiratory apnea at the functional residual lung capacity. Images were acquired in the transverse and sagittal planes, covering the entire thoracic cavity using a steady-state free precession sequence. Typical imaging parameters were: spatial resolution 1.1 × 1.1 mm, slice thickness 5 mm, slice gap 0 mm, repetition time 3.1 ms, echo time 1.6 ms, flip angle 60°, no ECG triggering, sensitivity-encoding factor 2.

### Image analysis

**Figure 1:**
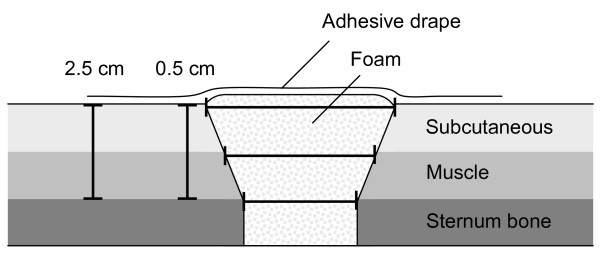
**Schematic illustration showing a transverse section through a sternotomy wound and the location of the wound dimension measurements**. The thick bracketed horizontal lines illustrate the lateral wound width at the level of subcutaneous tissue, muscle tissue and sternum bone. The thick bracketed vertical lines illustrate the anterior-posterior thickness of the soft tissue, including the muscle and subcutaneous tissue, at a lateral distance of 0.5 cm and 2.5 cm from the wound edge.

All images were evaluated using freely available software (Segment 1.699, available at http://segment.heiberg.se) [12]. Measurements of wound contraction and soft tissue macro-deformation were performed in the same transverse image at the cardiac midventricular level that were acquired before (0 mmHg) and after the application of -75, -125 and -175 mmHg. The distance between the two wound edges of subcutaneous tissue, muscle tissue and sternum bone were measured (lateral wound width). The anterior-posterior thickness of the soft tissue, including the subcutaneous and muscle tissue, was measured at a distance of 0.5 cm and 2.5 cm from the wound edge (Figure 1).

### Calculations and statistics

Statistical analysis was performed using paired Student's t-test. Significance was defined as p < 0.05. The results are presented as mean values ± the standard error of the mean (S.E.M.).

## Results

**Figure 2:**
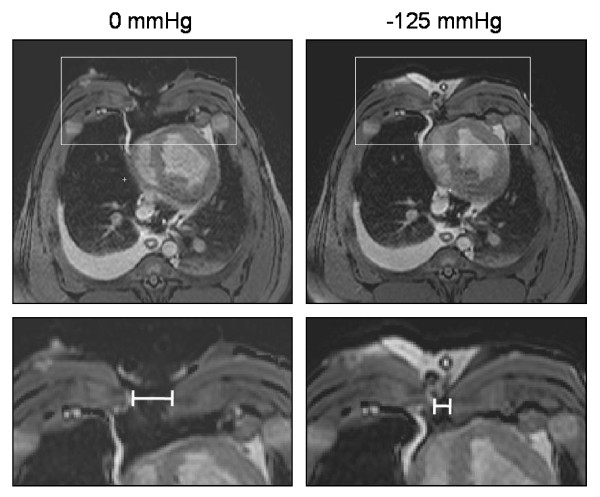
**Transverse magnetic resonance images at the cardiac midventricular level illustrating the wound contraction upon negative pressure wound therapy application**. The images were obtained before (0 mmHg) and after the application of -125 mmHg. The lower panels are enlargements of the insets in the upper panels and illustrate the position of the measurements taken. Note how negative pressure wound therapy pulls the two sternotomy wound edges closer together.

**Figure 3:**
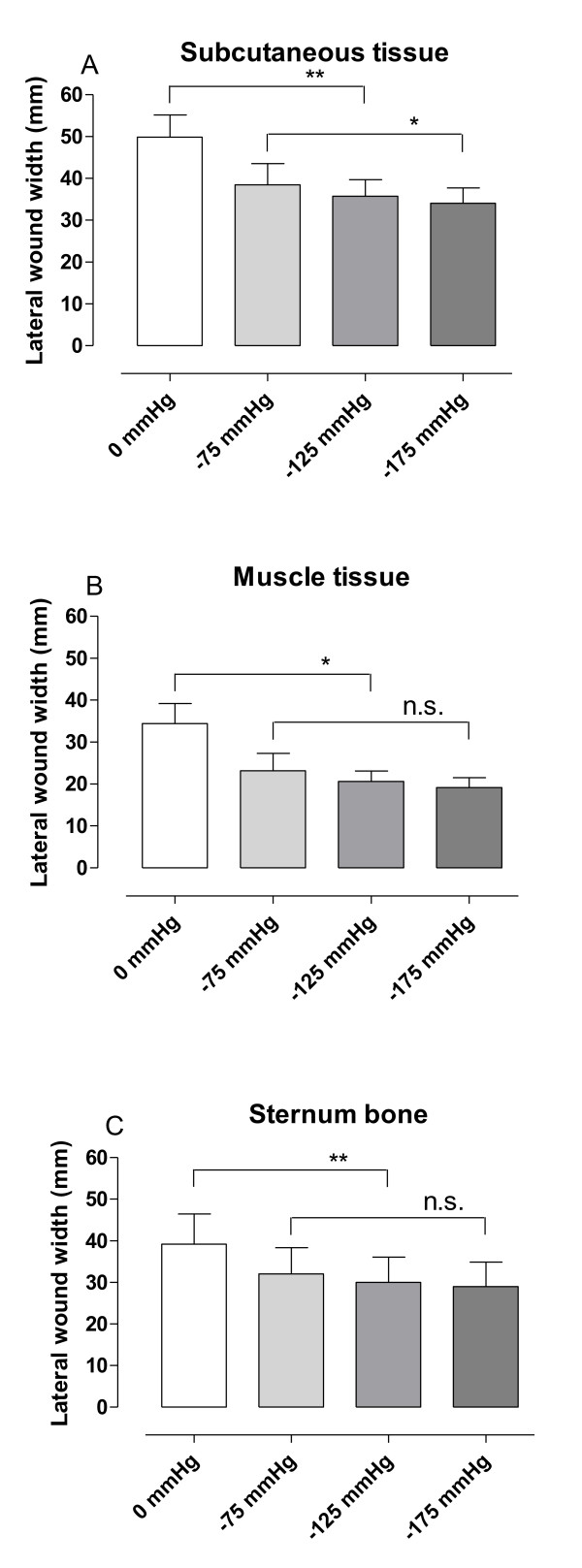
**Graphs showing wound contraction upon negative pressure application**. The distance between the wound edges (lateral wound width) in subcutaneous tissue (A), muscle tissue (B) and sternum bone (C), measured in transverse magnetic resonance images in sternotomized pigs before (0 mmHg) and after the application of negative pressure wound therapy (NPWT) at -75, -125 and -175 mmHg. Results are presented as mean values ± S.E.M. Statistical comparison was performed using Student's paired t-test. Significance is defined as p < 0.05 (*) and p < 0.01 (**) and n.s. denotes non-significance. Note the decrease in lateral wound width upon application of NPWT.

The sternotomy wound changed in appearance and the lateral wound width decreased when negative pressure was applied (Figure 2). The lateral wound width decreased from 39 ± 7 mm to 30 ± 6 mm, for sternum bone, upon application of -125 mmHg (p = 0.0027, n = 6, Figure 3). The greatest decrease in lateral wound width, as measured between the sternum bone edges, occurred when switching from 0 mmHg to -75 mmHg, and the level of negative pressure did not play a role for the degree of wound contraction (32 ± 6 mm at -75 mmHg and 29 ± 6 mm at -175 mmHg, for the sternum bone, p = 0.0897, n = 6, Figure 3).

The wound edge tissue was also deformed upon application of NPWT. The decrease in lateral wound width during NPWT was greater in subcutaneous tissue (14 ± 2 mm) than in sternum bone (9 ± 2 mm), which resulted in a ratio of subcutaneous to sternal decrease in wound width of 1.7 ± 0.3 (p = 0.0423), suggesting macro-deformation of the wound edge tissue. The major decrease in lateral wound width occurred when switching from 0 to -75 mmHg and the level of negative pressure did not play a significant role for the degree of wound contraction (23 ± 4 mm at -75 mmHg and 19 ± 2 mm at -175 mmHg, for muscle tissue p = 0.0982, n = 6, Figure 3).

**Figure 4:**
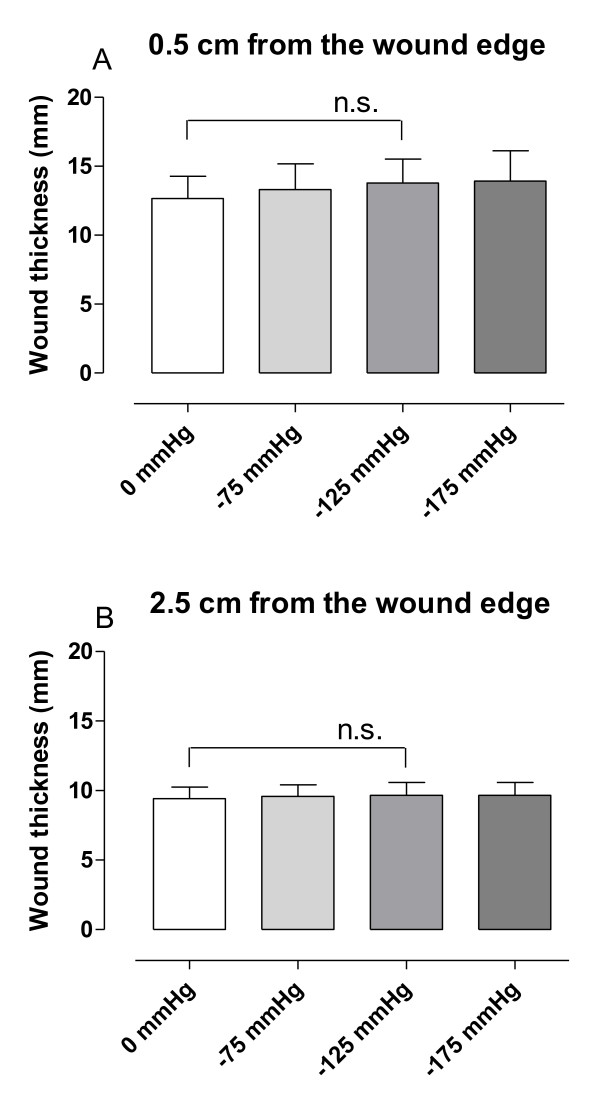
**Graphs showing anterior-posterior thickness of subcutaneous tissue and muscle tissue upon negative pressure application**. The anterior-posterior thickness of subcutaneous tissue and muscle tissue at 0.5 cm (A) and 2.5 cm (B) from the wound edge, measured in transverse magnetic resonance images in sternotomized pigs before (0 mmHg) and after the application of negative pressure wound therapy at -75, -125 and -175 mmHg. Results are presented as mean values ± S.E.M. Statistical comparison was performed using Student's paired t-test. Significance is defined as p < 0.05 and n.s. denotes non-significance.

The anterior-posterior thickness of the soft tissue, including subcutaneous and muscle tissue, at 0.5 and 2.5 cm laterally from the wound edge, was not affected by negative pressure (13 ± 2 mm at 0 mmHg and 14 ± 2 mm at -125 mmHg, 0.5 cm from the wound edge, p = 0.1111, n = 6, Figure 4).

## Discussion

The present study shows wound contraction upon application of NPWT in a porcine sternotomy wound model. Furthermore, it provides detailed evidence for the deformation of the wound edge tissue. Pulling forces by the negative pressure move the subcutaneous tissue wound edges together to a greater extent than the wound edges of the sternum bone. This presumably creates shearing forces in the tissue and at the wound-foam interface. This so called macro-deformation of the tissue is believed to be one of the fundamental mechanisms by which NPWT results in wound healing [11]. This mechanical effect of NPWT is thought to initiate a cascade of inter-related biological effects including the promotion of wound edge microvascular blood flow, removal of bacteria and stimulation of granulation tissue formation [7,10,13,14].

### Shearing forces at the foam-wound interface

Contraction of the wound and macro-deformation of the wound edge tissue upon NPWT, as shown in the present study, causes mechanical stress in the tissue. Mechanical stress is known to promote the expression of growth factors (e.g., vascular endothelial growth factor and fibroblast growth factor-2) and to stimulate granulation tissue formation and angiogenesis [15-17]. In a computerized model of negative pressure-induced wound deformation, most elements were stretched five to twenty percent by NPWT [11], which is similar to *in vitro *strain levels shown to promote cellular proliferation. The beneficial effects of NPWT on healing may depend on these macro-mechanical effects and the shearing forces at the foam-wound interface.

### Blood flow

The mechanical effect of NPWT on the wound edge tissue is also believed to alter microvascular blood flow. Close to the wound edge there is contraction of the tissue resulting in hypoperfusion [18-20]. Factors released in response to hypoperfusion are strong stimulators of angiogenesis and granulation tissue formation, which may be one of the mechanisms governing the positive effects of NPWT. Pressure against the wound wall may also be beneficial since it has been shown to tamponade superficial bleedings during surgical procedures [18] and reduce wound edge edema. Further away from the wound edge, microvascular blood flow is increased upon negative pressure application. It may be speculated that the pulling forces on the wound edge tissue opens up capillary beds and surges blood to the area. The present study shows differences in the wound edge tissue deformation when comparing subcutaneous and muscle tissue. Similarly, blood flow effects by NPWT are different in subcutaneous and muscle tissue [19,20]. It may be speculated that the mechanical effects that NPWT result in depend on the density of the tissue and the tissue composition of the treated wound.

### Sternum stability

In sternotomy wounds, there are underlying vital structures and an important aspect during treatment of these wounds is the heart and lung function and the reconstruction of a stable thorax. The present study shows that the sternotomy wound contracts during NPWT. This is in concordance with one of our previous studies showing that the sternum is stabilised and can withstand external forces during NPWT [21]. Stabilization of the sternum enables early mobilization which is crucial for the clinical outcome [22,23].

### Heart and lung function

As shown by the present study, NPWT contracts the wound and draws the two sternal edges together, thereby resealing the thoracic cavity. NPWT thus largely restores the macroscopic anatomical conditions in the thorax, which may explain the clinical benefits of NPWT over open-chest care, including reduced need for mechanical ventilation [24,25]. Sternotomy wound contraction and resealing of the sternum also has effects on the heart pumping function. The findings that cardiac output decreases during NPWT [26,27] have been a reason for concern. However, we now believe that cardiac output increases and the energy efficiency of cardiac pumping decreases upon sternotomy and both these measures return to pre-sternotomy levels when the thorax is resealed by NPWT[28]. It is reassuring to know that the effects on cardiac pumping function upon resealing of the thorax is physiological since many patients with deep sternal wound infections suffer impaired cardiac function and heart failure and may thereby be especially vulnerable to increased cardiac load.

### Different levels of negative pressure

In the present study, the greatest change in wound diameter was observed between 0 and -75 mmHg, and the level of negative pressure did not play a significant role for the degree of wound contraction. Similar findings were shown in a study by Isago et al [29], carried out in peripheral rat wounds and using polyurethane foam. Negative pressures of -50, -75 and -125 mmHg caused similar reduction in wound area. Furthermore, in a pig sternotomy wound model [21], the wound contraction upon NPWT application was similar in wounds treated with low (-50 to -100 mmHg) and high (-150 to -200 mmHg) negative pressures. Thus, both low and high levels of negative pressure will induce macro-mechanical deformation during NPWT.

## Conclusions

In conclusion, NPWT contracts the wound and causes macro-deformation of the wound edge tissue. This mechanical stress in the tissue and at the wound-foam interface creates shearing forces that is known to promote granulation tissue formation and facilitate healing.

## Competing interests

The authors declare that they have no competing interests.

## Authors' contributions

CT performed the image analysis, data analysis and drafted the manuscript. MU participated in the design of the study, image acquisition and analysis, data analysis and drafting the manuscript. HE participated in the design of the study and image acquisition. HA participated in the design of the study. RI participated in the design of the study and performed the surgical procedures. MM conceived of the study, participated in the surgical procedures, data analysis, drafting the manuscript and participated in its design and coordination. All authors critically revised the manuscript for important intellectual content, and approved the final manuscript.
